# Academic Help-Seeking Behaviours of Undergraduate Pharmacy Students in Saudi Arabia: Usage and Helpfulness of Resources

**DOI:** 10.3390/healthcare10071264

**Published:** 2022-07-07

**Authors:** Dalia Almaghaslah, Abdulrhman Alsayari

**Affiliations:** 1 Department of Clinical Pharmacy, College of Pharmacy, King Khalid University, Abha 61441, Saudi Arabia; 2 Department of Pharmacognosy, College of Pharmacy, King Khalid University, Abha 61441, Saudi Arabia; alsayari@kku.edu.sa

**Keywords:** academic help-seeking, pharmacy students, Saudi Arabia

## Abstract

Background: University students usually find it difficult to handle academic challenges by themselves and tend to seek help. Academic help seeking is defined as “a behavioral strategy that involves controlling one’s behavior and interacting socially to obtain help from other people.” Methods: A cross-sectional design was planned. An online self-administered questionnaire was used to collect data on academic help-seeking behaviours among year 5 undergraduate pharmacy students. Demographic and background information was described in terms of frequency. Their reasons for seeking academic help (five items) and factors considered for choosing academic resources (six items) used a Likert scale ranging from one (strongly disagree) to five (strongly agree). Usefulness (ten items) was rated on a five-point scale ranging from ‘very useful’ to ‘not useful at all.’ Frequency (ten items) was rated on a five-point scale ranging from ‘never’ to ‘every day.’ Results: A total of 116 pharmacy students completed the survey. The study indicated that the key reasons for seeking help were positive factors—a further improvement of one’s learning and the desire to complete a course. The resources that were most frequently used were peers, the course online portal, and online educational resources. The resources that students found most helpful were peers, the course instructor, textbooks, and online resources. Discussion and conclusion: The frequency of using a certain support resource was not mirrored by its helpfulness. Resources, including professors, teaching assistants, and textbooks, were found helpful but not frequently consulted for help. On the other hand, summaries and notes, for example, were frequently used but not particularly helpful. However, peers currently taking the course and the course management system were found to be very useful and frequently used.

## 1. Introduction

Pharmacy curricula have undergone core changes over the past decade as a result of changes in pharmacy practice that have been influenced by economics, politics, and social environments [1]. The expansion of the role of pharmacists has required student pharmacists to be well prepared at the undergraduate education levels [2]. Pharmacists are expected to have the required knowledge, attitude, and competency to be able to provide optimal pharmaceutical care [3,4]. As a result, pharmacy students experience a high academic workload, stiff competition, and high pressure to succeed [5].

Academic overload, along with socioeducational situations, concepts, and subjects, might sometimes exceed students’ capabilities, expectations, and abilities. Hence, it may expose students to stress and negatively impact their wellbeing and mental health [6]. University students usually find it difficult to handle these challenges by themselves and tend to seek academic help.

Academic help seeking is defined as “*a behavioral strategy that involves controlling one’s behavior and interacting socially to obtain help from other people*” [6]. Help seeking could be formal, where help is sought from those who have a degree in the relevant discipline, or informal, where help is sought from family members or friends [7]. In academia, formal help seeking includes bringing an inquiry into the classroom, as well as asking questions of the course instructor or academic student services outside the classroom, whereas informal help seeking happens when students ask questions of their classmates, peers, coworkers, or other instructors [8].

The literature has reported that pharmacy students avoid seeking formal academic help from their course instructors [9]. They are more likely to seek support on curricular and extracurricular opportunities from peers or student social networks [10]. Some factors that affect help-seeking behaviours are positive, such as having a high internal motivation level. Alternately, some factors are negative, such as the fear of having an undesirable experience as a result of seeking help, which then affects their self-esteem and causes social shame [6,9]. Other influences that affect academic help include students’ perceptions and beliefs, ego achievement goals, classroom norms, cultural and situational contexts, along with helper characteristics such as the instructor’s approachability and flexibility [6,9]. Academic help seeking has previously been reported to enable students to cope with academic challenges and is associated with academic success [9].

Pharmacy education requirements in Saudi Arabia have been influenced by international trends in terms of shifting to PharmD programmes, increasing the number of years of undergraduate programmes, education authorities imposing accreditations on pharmacy colleges, as well as the establishment of the Saudi pharmacists’ licensure examination (SPLE). All these factors have probably contributed to the increasing academic workload, the pressure to succeed, and the competition among undergraduate pharmacy students [11]. A previous study looked at mental-health-seeking behaviour among pharmacy students in Saudi Arabia [7]. However, no previous study has discussed academic help-seeking behaviours among undergraduate pharmacy students. The current study aimed to explore the reasons behind academic help seeking, assessing the resources available to assist students, the frequency of use, perceived usefulness, and factors considered when choosing a certain resource. This study took a broader approach and included academic help resources external to oneself, such as online resources, textbooks, peers’ notes, and summaries.

## 2. Methods

### 2.1. Study Design

The study used a cross-sectional design self-administered online questionnaire. The study was conducted between February and May 2022.

### 2.2. Settings

The study was conducted at a College of Pharmacy in Saudi Arabia between February and May 2022.

### 2.3. Participants

Year 5 (final year of didactic curriculum) undergraduate pharmacy students were selected as the study population. The inclusion criteria were as follows: undergraduate pharmacy program in year 5 aged ≥ 18 years. The exclusion criteria were as follows: students enrolled in undergraduate programs other than pharmacy, pharmacy students in years 1 to 4, and pharmacy students aged 17 years or younger.

### 2.4. Sample Size and Sampling Procedure

A nonprobability convenience sample was chosen. An invitation was sent to all students through the online management system (Blackboard). Two weeks after the initial invitation, a further reminder was sent through Blackboard. A final reminder was sent after one month. The reason for choosing this group of students was that they were exposed to different learning resources and experienced various academic help-seeking behaviours during the 5 years of their pharmacy programme. The total number of students enrolled in the course was (n = 140). Participation in the study was voluntary, but an extra mark was awarded to students who completed the survey. Participants were recruited through Blackboard announcements and direct in-person recruitment in classes. The sample size was determined based on the total number of students enrolled in year 5 (final year of didactic curriculum) (n = 140) and determined by using a Raosoft sample size calculator (http://www.raosoft.com/samplesize.html) (accessed on 15 March 2022) with a predetermined margin of error of 5% and a confidence level of 95%. In order to minimise erroneous findings and increase study reliability, the target sample size was set at 103 students.

### 2.5. Data Collection Form

The survey consisted of four sections. The first section collected participants’ demographic information, including age, gender, and GPA. The second section, asking the reasons for seeking academic help, was adopted from previous studies [9]. It contained 5 items which used a five-point Likert scale: (1) strongly disagree; (2) disagree; (3) neutral; (4) agree; and (5) strongly agree. The third section assessed the frequency and perceived usefulness of the resources; it was also adopted from a previous study with similar aims [9]. Additionally, frequency of use of the ten help-seeking resources was assessed using a five-point Likert scale: (1) never; (2) every few weeks; (3) once a week; (4) every few days; and (5) every day. The helpfulness of these ten help-seeking resources was assessed using a five-point Likert scale with the descriptors: (1) not useful at all; (2) not useful; (3) sometimes useful; (4) useful; and (5) very useful. The modifications that were determined in this section were based on informal discussions with students, who suggested adding extra academic help-seeking resources, including private tutors, which may have not been used by students in different contexts. Section four (6 items) assessed the factors students considered when choosing certain academic resources. This five-point Likert scale included: (1) strongly disagree; (2) disagree; (3) neutral; (4) agree; and (5) strongly agree.

Face and content validity was conducted by a group of experts in the field of pharmacy education. The questionnaire was available in both Arabic and English after assuring the accuracy of translation through back-translation conducted by the study authors, who are bilingual speakers of both languages. The final questionnaire was piloted with five year 4 pharmacy students to ensure the clarity of the language and of the questionnaire structure. The results of the pilot were not included in the final results. The final questionnaire was administered through a survey document in Blackboard.

### 2.6. Statistical Analysis

The collected data were downloaded, entered, and analysed using the Statistical Package for Social Sciences (SPSS) version 26.0 for Mac. Demographic and background information was described in terms of frequency. Reasons for seeking academic help (five items) and factors considered for choosing academic resources (six items) used a Likert scale ranging from 1 (strongly disagree) to 5 (strongly agree). The usefulness of resources (ten items) was rated on a five-point scale ranging from ‘very useful’ to ‘not useful at all.’ Frequency (ten items) was rated on a five-point scale ranging from ‘never’ to ‘every day’.

The distribution of the scale was presented in percentages, as well as the mean and SD. The internal consistency and reliability of the scales was assessed using Cronbach’s alpha coefficient, with the minimum recommended level being 0.70.

### 2.7. Ethical Consideration

An ethical clearance was given by the Ethics Committee at King Khalid University ECM# 2022-1904. Written informed consent was obtained from all participants. Taking part in the study was voluntary and potential participants had the right to decline the invitation without any penalties. The collected data were securely kept by the study authors. The study was carried out in accordance with the Declaration of Helsinki.

## 3. Results

Participants in the study included 116 students of the total 140 year 5 pharmacy students. Just under two-thirds (73 of participants) were female. More than two-thirds (67.3%) of them were between the ages of 22 and 23. Just under half of the participants (48.3%) had earned a GPA of >4 out of 5 (Table 1).

Table 2 shows the motives that encouraged students to seek academic help. The top reasons were a further development of their learning and the desire to successfully complete a course, with a mean of 3.9 out of 5. Other factors were a fear of failure, with a mean of 3.7 out of 5, and the perceived difficulty of the course, with a mean of 3.6 out of 5. Perceived faculty helpfulness scored the least, with a mean of 3.4 out of 5.

Table 3 shows comparisons between the frequency of use and the helpfulness of each academic resource. The only two resources that students both used frequently and found to be helpful were the course online portal, with a mean difference of 0.034, and peers currently enrolled in the class, with a mean difference of −0.18, where the *p* values indicated no statistically significant difference between frequency and helpfulness (*p* = 0.75 and 0.062, respectively). Resources, including the online resources specifically created for the course (mean difference = −0.465; *p* value = 0.001) and those not created for the course (mean difference = −0.75; *p* value =0.0001), the course instructor (mean difference = −0.84; *p* value = 0.0001), teaching assistant (mean difference = −0.92; *p* value = 0.0001), and peers not currently enrolled in the course (mean difference = −2.05; *p* value = 0.0001), were found to be helpful but not as frequently used. The only resource that students used frequently but found to be less useful was peers’ summaries or notes, with a mean difference of −0.89 and *p* value = 0.0001.

Table 3 and Table 4 show the frequency of use and the helpfulness of academic help-seeking behaviours. The course online portal, online resources, online resources specifically created for the course, online resources not specifically created for the course, peers currently enrolled in the course, and peer notes/summaries scored the highest frequencies among the resources, with means of 3.9, 3.2, 3.2, 4.2, 4.2, and 3.7 out of 5, respectively. The resources that were least frequently used were textbooks, private tutors, and other peers not currently enrolled in the course, with means of 1.8, 2.4, and 2.1 out of 5, respectively. On the other hand, the resources that scored highest in helpfulness were peers currently enrolled in the course, other peers not currently enrolled in the course, the course online portal, and online resources not specifically created for the course, with means of 4.2, 4.2, 3.9, and 3.9 out of 5, respectively. The resources that were found least useful were peer notes, teaching assistants, and private tutors, with means of 2.9, 3.4, and 3.5 out of 5, respectively.

Table 5 presents the factors that students considered when choosing help-seeking resources, including validity, accessibility, cost, and user-friendliness. Cost and easiness were also relevant, with means skewed towards five (strongly agree).

Table 6 presents the rate of agreement with the motives for seeking academic help: frequency, usefulness of resources, and criteria for choosing resources. These scales were treated as continuous variables, ranging from one (strongly disagree) to five (strongly agree) for both the motives and choosing resources criteria, ranging from one (not useful at all) to five (very useful) for usefulness, and ranging from one (never) to five (everyday) for frequency. The mean values of the overall agreement with help-seeking reasons were 3.7, 3, 3.6, and 4.2 for frequency, usefulness, and criteria for selecting resources, respectively. All scales had Cronbach alpha coefficients greater than 0.7, indicating interitem reliability.

## 4. Discussion

Pharmacy education has gone through tremendous changes globally. Educational requirements in Saudi Arabia have followed global trends in terms of shifting to PharmD programmes, increasing the number of years of undergraduate programmes, regulatory bodies imposing accreditations on pharmacy schools, as well as the establishment of the Saudi pharmacists’ licensure examination (SPLE). All these factors have probably contributed to an increasing academic workload, greater pressure to succeed, and higher competition among undergraduate pharmacy students. Students tend to seek academic help to survive university life and successfully earn a degree. This study was conducted to evaluate academic help-seeking behaviours among undergraduate pharmacy students in Saudi Arabia.

The current study found that positive factors, including the desire to complete a course and a further development of students’ learning, were among the top influences that encouraged participants to seek academic help, as compared to negative factors, such as a fear of failure and the perceived difficulty of the course.

The frequency of using academic help resources indicated that students often asked for support from their classmates more frequently than from any other resources, including formal resources. Previous studies reported that students often seek help from informal resources, i.e., classmates, rather than formal resources, i.e., teaching assistants and course instructors [9]. Other resources that students used quite frequently were online resources, including the course online management system and other online resources not created specifically for the course. The digitalisation of education, especially during the COVID-19 pandemic, has changed students’ preferences towards online education. A recent study concluded that Saudi pharmacy students valued the online management system for the delivery of course materials, as well as the use of other online resources, i.e., social media created specifically for the delivery of course content and extracurricular materials [12]. Another study confirmed that pharmacy students in a pharmacy college in Saudi Arabia had a feasible experience with online education during the COVID-19 pandemic, due to their competent online skills and their access to technology [13]. Textbooks, on the other hand, were the least frequently used. Similar findings were reported in a previous study that found that younger pharmacy students tended to use online resources and electronic textbooks more than printed textbooks to obtain drug information [14].

Interestingly, the resources that students found more helpful were not necessarily used frequently. For example, although students found the course instructor/teaching assistant to be helpful, they did not frequently ask for their help. This could be explained by the factors that students considered when reaching out for help, such as accessibility and approachability. This point was explained previously by Wirtz and colleagues, as they mentioned that peers are more accessible due to their physical presence in the same space, or through phone calls and group messaging applications, while teaching staff are harder to reach due to their busy schedules and limited office hours [15]. The same applied to colleagues who were not currently taking the course, as they were found to be helpful. However, they were not asked for help quite so regularly, perhaps due to different schedules that caused them to be less available. A study assessing Saudi students’ perceptions towards online education indicated that despite students’ preferences for online education in courses they perceived as less challenging, they still appreciated the value of having the course instructor physically present in face-to-face sessions [16].

Another example was students’ summaries/notes. Students indicated that they would use this resource frequently, although they did not find it very useful. This also could be explained by the fact that students considered peers’ notes less valid than the course instructor or a textbook; nonetheless, they are easily accessible.

The academic help resources that students scored highly in both frequency and helpfulness were peers currently enrolled in the course and the course online portal, considering that these resources, more or less, fulfilled all of the criteria that students valued in a resource, including accessibility, validity, user-friendliness, approachability, and easiness.

As a recommendation based on this study’s findings, faculty members should consider offering academic support by uploading online resources to the course online portal, creating online materials for the course by using social media, and suggesting valid already existing online resources such as trusted YouTube channels, Massive Open Online Courses (MOOCs) and online career certificates (e.g., Coursera) that support students’ learning. Online resources should be offered as an alternative to textbooks in course specifications. Students should be encouraged to communicate and support each other’s learning; colleges could create official channels that connect students who need academic support with those who are happy to provide it (10).

## 5. Limitations

The findings in the current study were subject to a number of limitations. First, the fact that more than two-thirds of the participants in this small study were female may have skewed the results. Hence, the results from male participants might have been underrepresented. Second, the findings were limited by using a cross-sectional design which could not establish causality. Another limitation was that the study was conducted at a single pharmacy college in Saudi Arabia, so the generalisability of the findings was limited to those with similar contexts. Additionally, a lack of anonymity might have caused response bias. Nonprobability convenient sampling might have limited the variety of the sample.

## 6. Conclusions

This study assessed academic help-seeking behaviours among undergraduate pharmacy students in Saudi Arabia. The study indicated that the prime motives for seeking help were positive factors: further improvement to one’s learning and the desire to successfully complete a course. The study also indicated that the frequency of using a certain support resource was not always mirrored by its helpfulness. Resources, including professors, teaching assistants, and textbooks, were found to be helpful but not frequently used. On the other hand, summaries and notes, for example, were frequently used but not particularly helpful. However, peers currently taking the course and the course management system were found to be both frequently consulted and useful. The factors that affected students’ choices when seeking help included validity, accessibility, cost, easiness, approachability, and user-friendliness. Therefore, students should be supported by pharmacy colleges to consult the most helpful and accessible resources. Interventions that aim to improve academic help-seeking behaviours should be a priority, such as strengthening student communication and peer support, encouraging faculty to be more approachable, and expanding online resources.

## Figures and Tables

**Table 1:** Demographics.

	Frequency	Percentage
**Gender**		
**Male**	43	37.1
**Female**	73	62.9
**Age**		
**21**	27	23.3
**22**	38	32.8
**23**	40	34.5
**≥24**	11	9.5
**GPA**		
**<2**	7	6
**2–3**	14	12.1
**3–4**	39	33.6
**>4**	56	48.3

**Table 2:** Reasons for seeking academic help.

Reason	Strongly Disagree	Disagree	Neutral	Agree	Strongly Agree	Skew	Mean	SD
	Frequency, Distribution of Responses (%)							
**Perceived difficulty of course**	8, 6.9	1, 0.9	42, 36.2	47, 40.5	18, 40.5	−0.8	3.6	0.9
**Further development and improvement in my learning**	2, 1.7	4, 3.4	24, 20.7	50, 43.1	36, 31	−0.8	3.9	0.9
**Fear of failure**	8, 6.9	12, 10.3	23, 19.8	33, 28.4	40, 34.5	−0.7	3.7	1.2
**Desire to complete a course; perceived academic competence**	4, 3.4	2, 1.7	26, 22.4	43, 37.1	41, 35.3	−0.9	3.9	0.9
**Perceived faculty helpfulness**	7, 6	6, 5.2	50, 43.1	37, 31.9	16, 13.8	−0.9	3.4	0.9

**Table 3:** Frequency of use of academic resources.

Resource	Never	Every Few Weeks	Once a Week	Every Few Days	Everyday	Skew	Mean	SD
**Online course portal (Blackboard)**	6, 5.2	6, 5.2	10, 8.6	55, 47.4	39, 33.6	−1.4	3.9	1.0
**Online resources specifically created for the course**	18, 15.5	20, 17.2	21, 18.1	37, 31.9	20, 17.2	−0.3	3.2	1.3
**Online resources not created specifically for the course** **(YouTube channel/Blogs/Twitter blogs)**	16, 13.8	27, 23.3	17, 14.7	30, 25.9	26, 22.4	−0.1	3.2	1.4
**Textbooks that are not recommended by the course instructor**	62, 53.4	24, 20.7	18, 15.5	5, 4.3	7, 6	1.2	1.8	1.9
**Teaching assistant in class or lab**	32, 27.6	36, 31	15, 12.9	19, 16.4	14, 12.1	0.5	2.5	1.4
**The course instructor/professor during class or lab**	22, 19	24, 20.7	21, 18.1	32, 27.6	17, 14.7	−0.07	2.9	1.4
**Private tutor**	45, 38.8	24, 20.7	15, 12.9	15, 12.9	17, 14.7	0.6	2.4	1.5
**Peers currently enrolled in course**	6, 5.2	7, 6	9, 7.8	48, 41.4	46, 39.7	−1.3	4	1.0
**Other peers not currently enrolled in course**	51, 44	26, 22.4	21, 18.1	8, 6.9	10, 8.6	0.9	2.1	1.3
**Peer notes/summaries**	12, 10.3	18, 15.5	13, 11.2	27, 23.3	46, 39.7	−0.2	3.7	1.4

**Table 4:** Helpfulness of academic resources.

Resource	Not at All Useful	Not Useful	Sometimes Useful	Useful	Very Useful	Skew	Mean	SD
**Online course portal (Blackboard)**	6, 5.2	4, 3.4	25, 21.6	35, 30.2	46, 39.7	−1.01	3.9	1.1
**Online resources specifically created for the course**	7, 6	8, 6.9	35, 30.2	35, 30,2	31, 26.7	−0.6	3.6	1.1
**Online resources not created specifically for the course (YouTube channel/Blogs/Twitter blogs)**	7, 5	5, 4.3	28, 24.1	23, 19.8	53, 45.7	−0.9	3.9	1.1
**Textbooks that are not recommended by the course instructor**	23, 19.8	15, 12.9	51, 44	18, 15.5	9, 7.8	−0.04	3.8	1.2
**Teaching assistant in class or lab**	8, 6.9	11, 9.5	36, 31	41, 35.3	20, 17.2	−0.5	3.4	1.09
**The course instructor/professor during class or lab**	5, 4.3	6, 5.2	24, 20.7	50, 43.1	31, 26.7	−0.9	3.8	1.02
**Private tutor**	18, 15.5	7, 6	32, 27.6	21, 18.1	38, 32.8	−0.4	3.5	1.4
**Peers currently enrolled in course**	4, 2	2, 1.7	17, 14.7	34, 29.3	59, 50.9	−1.4	4.2	0.9
**Other peers not currently enrolled in course**	5, 4.3	2, 1.7	14, 12.1	40, 34.5	55, 47.4	−1.5	4.2	1.01
**Peer notes/summaries**	12, 17.2	18, 15.5	46, 39.7	18, 15.5	14, 12.1	0.025	2.9	1.2

**Table 5:** Factors considered for choosing academic resources.

Criteria	Strongly Disagree	Disagree	Neutral	Agree	Strongly Agree	Skew	Mean	SD
	Frequency, Distribution of Responses (%)							
**Validity**	3, 2.6		15, 12.9	30, 25.9	68, 58.6	−1.7	4.4	0.9
**Accessibility**	0	3, 2.6	13, 11.2	35, 30.2	65, 56	−1.1	4.4	0.8
**Cost**	8, 6.9	4, 3.4	31, 26.7	28, 24.1	45, 38.8	−0.8	3.8	1.2
**User-friendliness**	0	4, 3.4	19, 16.4	33, 28.4	60, 51.7	−0.9	4.3	0.9
**Easiness**	2, 1.7	3, 2.6	23, 19.8	29, 25	59, 50.9	−1.1	4.2	0.9
**Approachability**	3, 2.6	4, 3.4	17, 14.7	32, 27.6	60, 51.7	−1.3	4.2	0.9

**Table 6:** Distribution and internal consistency of help-seeking reasons and criteria for selecting resources.

Description of Scale	≤1	≤2	≤3	≤4	≤5	Skew	Mean	SD	Cronbach α
**Help-seeking reasons**	0.9	1.7	18.1	69.8	100	−0.9	3.7	0.7	0.7
**Frequency**	0	0	56.9	93.1	100	−0.5	3	0.7	0.7
**Usefulness**	0.9	3.4	11.2	72.4	100	−1.2	3.6	0.6	0.8
**Criteria for selecting resources**	0	0	11.2	44	100	−0.65	4.2	0.7	0.8

## Data Availability

The data presented in this study are available on request from the corresponding author.
